# Facilitating Eudaimonic Well-Being in Mental Health Care Organizations: The Role of Servant Leadership and Workplace Civility Climate

**DOI:** 10.3390/ijerph17041173

**Published:** 2020-02-12

**Authors:** Susan der Kinderen, Amber Valk, Svetlana N. Khapova, Maria Tims

**Affiliations:** School of Business and Economics, Vrije Universiteit, De Boelelaan 1105, 1081 HV Amsterdam, The Netherlands; amber.valk93@gmail.com (A.V.); s.n.khapova@vu.nl (S.N.K.); m.tims@vu.nl (M.T.)

**Keywords:** eudaimonic well-being, psychological well-being, well-being at work, servant leadership, workplace civility climate, health care management

## Abstract

Demanding and complex work within mental health care organizations places employee well-being at risk and raises the question of how we can positively influence the psychological well-being and functioning of these employees. This study explores the role of servant leadership and workplace civility climate in shaping eudaimonic well-being among 312 employees in a Dutch mental health care organization. The findings showed that servant leadership had a stronger relationship with eudaimonic well-being when workplace civility climate was high. Furthermore, the results showed that servant leadership was positively related to workplace outcomes, partially through eudaimonic well-being, and that this mediating process varied across different levels of workplace civility climate. This study contributes to the scholarly understanding of the role of servant leadership and a positive work climate in shaping psychological well-being at work.

## 1. Introduction

“Put on your own oxygen mask before assisting others”—a strong metaphor when applied to mental health care organizations where the impact of relentless change, the high incidence of burnout, and rapid turnover rates threaten to compromise the quality and safety of patient care [1]. The need to attract, retain and sustain a vital, flourishing, and productive workforce is critical—both for the well-being of the professionals themselves, and for sustaining organizations in their safe and meaningful service to a society burdened by escalating health care needs [2,3]. This striving for organizational performance, without a cost to employee health and society, has given renewed impetus to the positive well-being at work literatures. It encourages new perspectives on the role of the organizational context, particularly in terms of more relational leadership styles and a positive work climate, which could serve to navigate this challenge and facilitate gain in both employee well-being, as well as work-related outcomes [4]. Most of what we know about positive psychological well-being at work has been captured by hedonic conceptualizations of subjective well-being, such as positive and negative affect, job satisfaction, and happiness [5]. This conceptualization of well-being has been described as promoting a short-term, passive state that does not equate with the need to be future-focused, adaptive, innovative, or in a growth state [6]. Eudaimonic well-being (EWB) extends our definition of positive psychological well-being by capturing existential and motivational elements of good functioning such as having purpose in life, personal growth, positive relationships, and the true expression of self [7,8,9,10]. In a context where the well-being of our workforce plays a critical role in sustaining a delicate economic, social, and political balance, it seems prudent to activate all potential avenues towards well-being, not only in hedonic terms of happiness and satisfaction, but also in terms of (EWB) which goes beyond feeling good to capture psychological good functioning [11]. This study aims to contribute to our limited understanding of the unique contribution of EWB—both in terms of how it is shaped, as well as to how, and under which conditions, it relates to work-related outcomes [11,12]. 

Using the framework for health service organizations suggested by Michie and West [2], we propose a model which includes contextual factors, people management, EWB, and work-related outcomes. While there is evidence that EWB, as a largely cognitive and behavioral construct, is particularly open to being shaped via individual, as well as contextual influences [8], a recent review on leadership behavior and employee well-being highlights a need for more conclusive insights into how leadership behaviors relate to eudaimonic well-being and into the role of moderator variables, which may create boundary conditions for this relationship [13]. As the way organizations gain competitive advantage has evolved from increasedproduction and efficiency, to providing unique services and knowledge, and finally to providing meaningful experiences and social networks [14], so there is a shift in the power dynamics from institution to individual, which requires a shift in leadership style from authoritarian control to one which supports intrinsic motivation and fulfillment [15]. Recognizing that health care organizations work “both in society and for society” [16] (p. 198) suggests the need for an ethical, employee-focused leadership which, alongside organizational goals, places value on employee well-being [17]. Where transactional leadership focuses on effectively achieving organizational objectives, a servant leader is defined as a leader who chooses to serve the priorities and growth of the other, helping followers achieve their full potential [18]. Due to the inherently servant-like nature of the health care sector it is suggested that a more servant-oriented leadership style is conducive to enhancing both employee well-being, as well as organizational outcomes [19,20]. We examine the role of servant leadership, which focuses on leaders developing and nurturing followers’ strengths and skills, as a key antecedent influencing both individual EWB, as well as positive work-related outcomes among mental health care workers. 

Although leadership studies have established the role of leadership in contributing towards followers’ mental health and positive workplace outcomes [21], leadership behavior at work cannot be understood in isolation. Contextual factors will either promote or constrain behaviors and thereby shape leadership’s influence on outcomes such as followers’ well-being [22,23]. The role of leadership, well-being, and examinations of contextual conditions moderating this relationship are rare [13,21,22,24]. There is evidence for the role of psychosocial safety climate, defined as those policies, practices, and procedures which aim to protect workers psychological health, moderating the relationship between job demands and health outcomes [25]. However, we examine at the more proximal role of workplace civility climate (WPCC), as embodied by the workgroup itself, and in which this leader–follower relationship is embedded [26]. We expect this WPCC to have a conditional interactive effect on the relationship between servant leadership, EWB, and the positive workplace outcomes. 

The traditional proposition that a happy worker is a productive worker [27] has shown mixed results, suggesting that this relationship is mediated by an interplay of variables such as relationships, health, and motivation [28]. Moreover, the focus on happiness, job satisfaction, or work engagement, as mechanisms to improving organizational performance, could be at the cost of personal psychological well-being and functioning of employees [4,29,30]. In a sector where high work demands can compromise performance and patient safety [19], and where ongoing change applies pressure to innovate and perform, EWB offers a comprehensive explanation for how individual functioning, through these eudaimonic elements of personal growth, positive relationships, and purpose, can impact positive organizational outcomes. There is evidence that EWB is permeated by the work activities we engage in and is related to a number of positive outcomes such as job performance, employee commitment, and subjective well-being [31,32,33,34]. For the purpose of this study we have chosen to examine three workplace outcomes which we believe would be relevant within a health care setting: (1) Task performance [35] and (2) innovative work behavior [36], which act as performance indicators in an industry that is regularly challenged with high workload, constant change, staff shortages, and pressure for increased efficiency [1], and (3) work engagement [37], as the antipode to burnout, which is related to employee commitment and job satisfaction [38]. 

This study makes a number of contributions to positive workplace well-being regarding how organizational factors and context influence psychological functioning and well-being of employees, while simultaneously contributing to workplace outcomes. First, we bring the eudaimonic component of positive employee well-being into organization studies hereby emphasizing the role of the workplace in eliciting our domain-free full psychological functioning. Second, we identify the specific role of servant leadership, under the condition of a civil work climate, in impacting not only our well-being but as a leadership style which contributes to our workplace performance through EWB. We conclude with a discussion of this study’s contributions regarding how organizational factors and context influence the positive psychological functioning and well-being of employees, while simultaneously contributing to workplace outcomes.

### 1.1. Theoretical Background and Hypotheses

For the purpose of this study, we define eudaimonic well-being as the process of realizing one’s full potential and being fully functioning in the face of life’s roles and challenges. This full functioning includes cognitive components, such as acting in line with deeply held values, having purpose and meaning in life, and behavioral elements such as seeking personal growth and building positive relationships [9,39,40]. Particularly in a healthcare context, where employees have consciously chosen to enter a caring profession, bringing personal values and norms into the workplace to provide a service to heal patients [41], we choose a definition of well-being which captures elements of personal growth, positive relationships, and purpose. Drawing on humanistic and human development theories Carol Ryff [9] defined a comprehensive model of eudaimonic well-being which includes six dimensions: Having a positive evaluation of oneself and one’s past (self-acceptance), having quality relationships with others (positive relations with others), continued growth, development and expansion of potential (personal growth), the belief that one’s goals are purposeful and meaningful (purpose in life), the ability to effectively and competently manage one’s life (environmental mastery), and finally, a sense of being self-determining, independent and regulating behavior from within (autonomy) [9]. This conceptualization, and the related scales, has been used widely in empirical studies—with a number of consolidations made by the author [42,43], but has made limited inroads into organizational studies. 

The conservation of resources theory (COR) is an integrative stress theory which explains how both environmental and internal processes impact our well-being [44]. The COR theory emphasizes the role of resources which are defined as those personal characteristics or conditions that are valued by the individual because of their function to help manage stress and gain or protect resources [45]. A key premise of the COR theory is that individuals are motivated to gain resources, resulting in a generation of resource gain [45]. Conceptually, EWB captures a number of what Hobfoll considered key, proximal, personal resources such as self-acceptance, feeling that life has meaning or purpose, a sense of mastery of one’s life and building positive relationships [46]. Following on this principle of resource gain we argue that these personal resources, in the form of EWB are shaped by workplace, contextual resources. 

#### 1.1.1. Servant Leadership, Workplace Civility Climate, and EWB

How these personal resources are supported by exposure to contextual, job-related resources and conditions can be explained through the lens of the job demands-resources (JD-R) model [47,48]. The JD-R theory posits that job resources, those physical or social aspects of a job that help achieve goals or stimulate personal growth, influence outcomes through an intrinsic or extrinsic motivational process [49]. COR and JD-R theories have previously been combined to explain the role of personal resources in the relationship between job resources and workplace outcomes [49,50]. Building on COR theory, Brummelhuis and Bakker [50] created a categorization of resources in which they distinguished between contextual and personal resources, where contextual resources ranged from objective conditions to social support, and personal resources from intrinsic energies such as mood to constructive resources such as knowledge or mental resilience. In a work setting, leadership could be seen as a resource falling in the category of contextual social support. The positive relationship between perceived supervisor support [51], transformational leadership [52], leader’s spiritual values [53], and EWB suggests that leaders who model serving behaviors, meaningful work, and the use of individual strengths [54] could shape EWB for health care workers. Servant leadership is recognized as a leadership style which benefits organizations, and society, through its focus on valuing, developing, and engaging employees in an authentic way [55]. In this study, we utilize a multi-dimensional model of servant leadership which includes seven dimensions: Emotional healing, empowering, creating value for the community, having conceptual skills, helping subordinates grow and succeed, putting subordinates first, and behaving ethically [20]. With its focus on facilitating autonomy, moral development and needs satisfaction of followers, this definition echoes many eudaimonic elements such as personal growth, purpose, and positive relationships which, in the case of a health care context, are aimed at serving the needs of the follower so as to serve a patient community beyond their own. We therefore argue that servant leadership, as a contextual job resource, should extend the pool of existing personal resources, as reflected by EWB [50]. Hence, we propose that:

**Hypothesis** **1.** *Servant leadership is positively related to the EWB of mental health care workers.*

While most studies have focused on the role of workplace incivility moderating between work experiences and mental strain [56,57], we propose that in a workplace where interaction is guided by strong civility norms this creates a condition under which servant leaders’ empowerment of followers is able to influence well-being [23]. The perception of civility implies that colleagues can be counted on to embrace the same unspoken rules for polite, respectful behavior and that they will actively uphold these norms in the group. This social climate becomes a fruitful psychosocial condition in which the resource gain, as explained by the COR theory, can take place [50]. In this respect, we propose that the presence of workplace civility norms would act as an enhancer moderator, and that high levels of WPCC would enable the ability of servant leaders to promote EWB [22]. Conversely, when there is low perceived WPCC, and rude behavior seems to have no consequences, it seems likely that the ability of a servant leader to role model positive, authentic behavior or to support follower development could be perceived as insincere or incoherent, and therefore inhibit the relationship to EWB. Hence, we propose the following:

**Hypothesis** **2.** *Workplace civility norms moderate the relationship between servant leadership and EWB such that the positive relationship will be stronger when workplace civility norms are high.*

#### 1.1.2. Servant Leadership, Positive Workplace Outcomes, and the Role of EWB 

It is acknowledged that health care employees actively bring the values, norms, and strengths related to providing a caring service into a highly demanding, professional workplace [41]. Previous studies have shown that challenging work conditions can facilitate the impact of personal resources on work engagement [58]. Building on the resource gain premise of the COR theory [46] and the motivational process of JD-R [47], which asserts that individuals with access to greater resources would be more capable of resource gain, we propose that when individuals experience EWB they activate the motivational process towards goal achievement and performance. Task performance, as a key element of job performance, is defined as employee behaviors which exhibit confidence in performing key job tasks or goals [59]. In studies using multi-dimensional, eudaimonic conceptualizations of well-being we find evidence for a relationship with contextual performance [60], job performance [61], and task-oriented personal initiative [62]. The eudaimonic process of becoming the best version of one’s self suggests the active use and development of strengths in a meaningful way [63]. Previous research has, albeit cautiously, linked servant leadership to task performance through the satisfaction of the needs for autonomy and competence [64]. It therefore follows that EWB could similarly elicit active contribution to organizational performance through the fulfillment of key tasks and services. 

Innovative work behavior is defined as individual behavior aimed toward the creative exploration, generation, promotion, and implementation of new ideas [36]. Several studies indicate the role of positive, affective well-being in creative and innovative work behavior [11,65]. However, we would argue that some elements of innovation are more likely to involve goal striving, struggle, and challenge, which are not necessarily associated with short-term happiness or positive affect, but to more eudaimonic feelings, such as interest and challenge [66].

Work engagement is defined as a work-related, positive, fulfilling state of mind characterized by vigor, dedication, and absorption [38]. Since the health care sector is renowned for high levels of burnout it is important to understand all avenues through which its antipode, work engagement, can be achieved. In line with the JD-R theory personal resources, captured by EWB, would relate to positive work outcomes such as work engagement [49]. Hence, we propose:

**Hypothesis** **3.** *EWB is positively related to (a) task performance, (b) innovative work behavior, and (c) work engagement.*

The position of leadership as an organizational success factor, as well as a key occupational health factor [21] suggests that EWB should play a mediating role between leadership and performance. Drawing again on the COR and JD-R theories this motivational process from job resources, to personal resources back to job resources, results in a resource gain cycle [45,49]. For example, should a servant leader model and support the learning and acquisition of planning as a new skill, the follower experiences this as EWB in terms of personal development and environmental mastery, which is then exhibited in the work domain as task performance. The relationship between leadership and workplace outcomes can be explained by a number of mechanisms, for example job stress, and not only domain-free EWB [21]. As such, it is proposed that EWB will only partially mediate this relationship. However, the role of WPCC, which we position as a conditional job resource, would continue to impact the strength of this relationship. Ten Brummelhuis and Bakker [50] position cultural values and social context as a macro resource which can moderate the relationship between contextual and personal resources. Following this reasoning, we argue that a high WPCC can serve as fertile ground for the servant leader to influence workplace outcomes through well-being. When a workplace context supports satisfaction of the basic needs of autonomy, competence, and relatedness the likelihood of more autonomous motivations in employees increases and contributes to high performance work behaviors, as well as to health and well-being [67]. For example, in a work context where there is an established norm of treating everyone with respect, one could expect increased access to resources, such as the supportive relationship of a colleague or leader, which would in turn impact task performance. When WPCC is low we could assume, for example, that aggressive outbursts are ignored and the ability of a servant leader to influence innovative work behavior through EWB, in terms of self-acceptance or positive relationships, would be limited. This moderated mediation [68,69] suggests that, depending on the level of WPCC, servant leadership would have a different relationship to workplace outcomes through EWB. Therefore, we propose the following:

**Hypothesis** **4.** *The mediation mechanism between servant leadership, EWB, and (a) task performance, (b) innovative work behavior and (c) work engagement, is moderated by WPCC such that this mediation is stronger for individuals high in WPCC than those low in WPCC.*

## 2. Materials and Methods

### 2.1. Participants and Procedure

This study was conducted in The Netherlands where ethical approval is only obligatory if the study falls under the Medical Research Involving Human Subjects Act (WMO). This study contained no medical elements, therefore requiring no ethics approval, which was confirmed with the university’s research office.

To conduct this research, self-reports and a correlational cross-sectional survey design were used. The invited, targeted sample consisted of all 1200 employees of a large mental health care institution in The Netherlands. Employees received an e-mail from their HRM department with an invitation to participate anonymously in the voluntary, online questionnaire which was made available for three weeks. Incomplete surveys (*n* = 154) were excluded, resulting in 312 subjects in the final sample (26% response rate). This sample was comprised of 28% males (M = 50.76 years, SD = 10.40), and 72% females (M = 45.21 years, SD = 12.10) with a mean age of 46.76 years (SD = 11.90). Working experience varied between 0.50 and 54 years (M = 25.34 years, SD = 12.77), and the average tenure was 16.07 years (SD = 12.12), ranging from a few months to 43 years. The majority of the respondents had completed a higher vocational or university education (65.7%). The sample population, in terms of gender and education levels, can be considered representative of the mental healthcare sector in The Netherlands [70]. It is worth noting that the survey invitation was extended to all employees in the organization and therefore included employees with direct patient contact, as well as managers and administrative staff. 

### 2.2. Measures

All measurement instruments are previously validated scales adapted into a five-point Likert scale (EWB, servant leadership, and WPCC: 1 = strongly disagree, 5 = strongly agree; work engagement, innovative work behavior, and task performance: 1 = never, 5 = always). Demographic variables including age, gender, education, work hours per week, work experience, and job tenure were collected through single item questions.

EWB was measured using a validated Dutch version [71] of Ryff’s scale of psychological well-being [9]. The scale consists of six dimensions: Personal growth, adapted to six items, example item ‘In general, I feel that I continue to learn more about myself as time goes by’, (α = 0.70); positive relations with others, six items, example item ‘I feel like I get a lot out of my friendships’, (α = 0.80); self-acceptance, six items, example item ‘I like most aspects of my personality’, (α = 0.77); autonomy, eight items, example item ‘I have confidence in my opinions, even if they are contrary to the general consensus’ (α = 0.75); environmental mastery, six items, example item ‘In general, I feel I am in charge of the situation in which I live’, (α = 0.72), and purpose in life, six items, example item ‘I enjoy making plans for the future and working to make them a reality’, (α = 0.71). A composite score of all 38 items indicated that the reliability of the total scale was very good (α = 0.91).

Servant leadership was measured with the shortened servant leadership scale developed by Liden et al. [72] and consisted of seven items covering the seven dimensions of servant leadership: Emotional healing; creating value for the community; conceptual skills; empowering; helping subordinates grow and succeed; putting subordinates first, and behaving ethically. An example item is ‘My manager makes my career development a priority’ (helping subordinates grow and succeed). The questions were translated into Dutch using the process of back translation. The reliability of the scale was Cronbach’s α = 0.82. 

WPCC was measured using the Civility Norms Questionnaire-Brief (CNQ-B) which covered employees’ experience of workgroup civility norms [73]. The scale consisted of four items, example items included ‘Rude behavior is not accepted by your coworkers’ and ‘Your coworkers make sure everyone in your unit/workgroup is treated with respect’. These items were translated into Dutch with the process of back translation. Cronbach’s alpha in this sample was α = 0.84. 

Task performance was measured using the task performance dimension from the Dutch version of the Individual Work Performance scale [35]. The Task Performance Scale consists of five items; an example item is ‘I was able to plan my work so that I finished it on time’. The reliability of the scale was Cronbach’s α = 0.80. 

Innovative work behavior was measured using the Innovative Work Behavior scale developed by De Jong and Den Hartog [36]. The ten items were translated into Dutch using the process of back translation. An example item is: ‘I wonder how things can be improved’. In this sample, the reliability of the scale was Cronbach’s α = 0.91. 

Work engagement was measured using the three-item version of the Utrecht Work Engagement Scale [37] comprising the dimensions: Vigor, dedication, and absorption. An example item was ‘At my job, I feel strong and vigorous’ (vigor). Reliability analysis showed that Cronbach’s α was 0.81. 

### 2.3. Data Analyses

We conducted confirmatory factor analyses (CFA) to assess the construct validity of the measurement scales: Servant leadership, workplace civility climate, eudaimonic well-being, work engagement, innovative work behavior, and task performance. CFA was conducted using the AMOS software package version 25 [74], where χ^2^/*df* values of less than 2.5, Tucker-Lewis index (TLI) and comparative fit index (CFI) values greater than 0.9, and the root mean square error of approximation (RMSEA) values less than 0.08, were considered indicative of an acceptable or good model fit [75,76]. The regression analyses were conducted with the program Statistical Package for the Social Sciences (SPSS) version 25. A significance level of α = 0.05 was used to test the hypotheses. Before testing the hypotheses, the correlations between all constructs were examined (Table 1). To test the hypotheses, several regression analyses using PROCESS macro models 1 and 4 and a moderated mediation analysis (by creating centered variables and the use of the macro PROCESS model 8) were conducted [69,77].

## 3. Results

### 3.1. Descriptive Statistics and Confirmatory Factor Analyses

Table 1 presents the means, standard deviations, and correlations for key study variables. EWB was significantly positively correlated with all study variables except for the demographic variables and WPCC. Servant leadership was significantly positively correlated with most study variables and negatively to gender and age. Innovative work behavior was significantly correlated to gender, tenure, work hours per week, and age. For all analyses with innovative work behavior as an outcome variable we controlled this effect. 

We conducted confirmatory factor analyses (CFA) on the full measurement model where variables servant leadership, workplace civility climate, eudaimonic well-being, work engagement, innovative work behavior, and task performance were modeled with their respective items, except for EWB where we used the six subscale means, as indicators of the latent variable. All standardized factor loadings were significant and loaded substantially on their latent factor (factor loadings ranged between 0.34 and 0.92, all *p* values < 0.001). The initial model fit was inadequate (χ^2^ = 1158.23, *df* = 545, CFI = 0.88, TLI = 0.87, RMSEA = 0.06). We examined the modification indices and added a covariance between two items of the WPCC scale, and two covariances between items of the innovative work behavior scale. On closer inspection, these item pairs were similarly worded, for example in the WPCC scale, ‘Rude behavior is not accepted by your coworkers’ and ‘Angry outbursts are not tolerated by anyone in your workgroup’. After these covariances were included, the measurement model showed a better fit to the data: χ^2^ = 954.32, *df* = 542, CFI = 0.92, TLI = 0.91, and RMSEA = 0.05, ∆*df* = 3, ∆χ^2^/∆df = 203.91/3, *p* < 0.001. Given that we only used existing, validated scales, we conclude that the measurement model supports the convergent validity of the constructs. 

### 3.2. Hypotheses Testing

Regression analyses were conducted to test the hypotheses. The findings provided support for the first hypothesis stating that servant leadership was positively related to EWB (β = 0.12, *p* = 0.03, and F = 4.72, *p* = 0.03). To test the second hypothesis a moderation analysis (PROCESS macro, model 1) was conducted using centered mean scores to compute an interaction term representing the interaction between servant leadership and WPCC (see Table 2). As can be seen in step 2 the main effect of servant leadership on EWB was significant (B = 0.06, *p* = 0.05) whereas the main effect of workplace civility on eudaimonic well-being was not (B = 0.03, *p* = 0.10). As hypothesized, the interaction effect between WPCC and servant leadership was positive and significant (β = 0.23, *p* < 0.001). 

**Figure 1:**
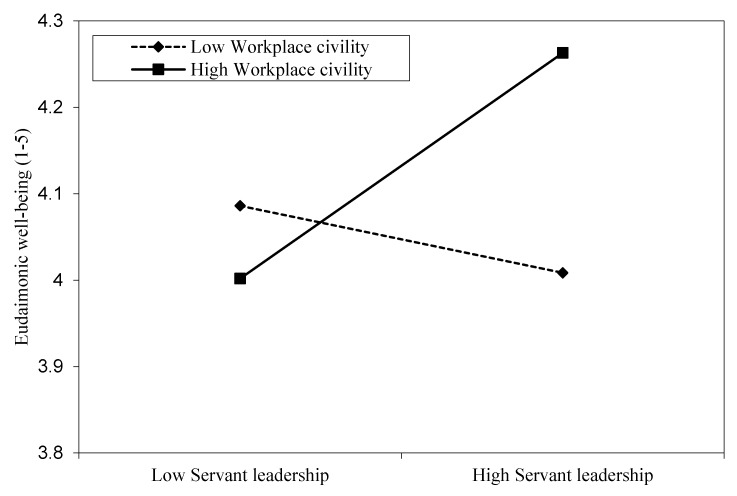
The moderating role of workplace civility in the relationship between servant leadership and EWB.

Figure 1 illustrates the interaction based on the low (1 SD below the centered mean) and high (1 SD above the centered mean) values of workplace civility. As can be seen in Figure 1, the relationship between servant leadership and EWB is stronger when workplace civility is high (simple slope = 0.16, *p* < 0.01) as opposed to when it is low (simple slope = −0.01, *p* = 0.77). This finding provides support for hypothesis two. 

The third hypothesis was also confirmed with EWB positively and significantly related to task performance (β = 0.39, *p* < 0.001), innovative work behavior (β = 0.15, *p* < 0.01), and work engagement (β = 0.39, *p* < 0.001).

Finally, the moderated mediation model in which WPCC moderates the mediation between servant leadership, EWB, and each of the three workplace outcomes was examined. Using the PROCESS macro and following the steps outlined by Preacher et al. [77], we conducted a mediation analysis [68] of servant leadership, through EWB, on task performance, innovative work behavior, and work engagement. As reported above (hypothesis 1), the first step results showed that servant leadership is positively and significantly related to EWB. Second, EWB is positively and significantly related to each of the three work-related outcomes (hypothesis 3). Furthermore, servant leadership is positively and significantly related to all three workplace outcomes: Task performance (β = 0.22, *p* < 0.01), innovative work behavior (β = 0.26, *p* < 0.01), and work engagement (B = 0.45, *p* < 0.01). In the final step, servant leadership and EWB were entered simultaneously into the model predicting the workplace outcomes. The results show a significant, positive indirect effect between servant leadership and task performance, via EWB (standardized indirect effect = 0.05, BCa CI [0.004, 0.09]). The indirect effect of servant leadership on innovative work behavior, through EWB, was positive and significant (standardized indirect effect = 0.02, BCa CI [0.001, 0.04]). The indirect effect of servant leadership on work engagement, through EWB was positive and significant (standardized indirect effect = 0.04, BCa CI [0.002, 0.09]). Therefore, the relationship between servant leadership and task performance, innovative work behavior, and work engagement, was partially mediated by EWB. 

The second condition for moderated mediation tested whether the interaction between servant leadership and WPCC was significant in predicting EWB. The results of the moderated regression are shown in Table 2 and provide support for the relationship between servant leadership and EWB being moderated by WPCC. The third condition in the moderated mediation analysis shows the conditional interaction effect between servant leadership and WPCC, on the relationship between servant leadership, mediated by EWB, and the three workplace outcomes. The results, reported in Table 3, show that for task performance there is moderated mediation (index of moderated mediation = 0.07, BCa CI [0.03, 0.12]). Specifically, when WPCC is high, EWB mediates the relationship between servant leadership and task performance (conditional indirect effect = 0.09, BCa CI [0.04, 0.14]). When workplace civility is low, there is no mediation between servant leadership, EWB, and task performance (conditional indirect effect = −0.01, BCa CI [−0.05, 0.03]). For work engagement there is moderated mediation (index of moderated mediation = 0.08, BCa CI [0.04, 0.13]). Specifically, when WPCC is high EWB mediates the relationship between servant leadership and work engagement (conditional indirect effect = 0.09, BCa CI [0.04, 0.16]). When WPCC is low there is no mediation between servant leadership, EWB, and work engagement (conditional indirect effect = −0.01, BCa CI [−0.05, 0.04]). For the outcome of innovative work behavior there is moderated mediation (index of moderated mediation = 0.03, BCa CI [0.01, 0.07]). Specifically, when WPCC is high, EWB mediates the relationship between servant leadership and innovative work behavior (conditional indirect effect = 0.04, BCa CI [0.01, 0.08]). When WPCC is low, there is no mediation of EWB between servant leadership and innovative work behavior (conditional indirect effect = 0.00, BCa CI [−0.02, 0.02]). 

## 4. Discussion

This study investigated the role of servant leadership and a civil work climate in shaping EWB in mental health care organizations. It also examined the effect of the interaction, between servant leadership and WPCC on three workplace outcomes through EWB. Our findings contribute to the literature on leadership and employee health in several ways.

First, we contribute to empirical studies on servant leadership in a health care setting by showing that servant leaders, who support the attainment of organizational objectives through facilitating the growth and potential of employees, impact followers EWB [20]. Second, this study extends our understanding of how servant leadership impacts well-being by providing initial evidence for WPCC as a contextual boundary factor which significantly impacts the power of this relationship. We expand on previous studies [56,73] by illustrating that the environment in which leaders and employees operate is important. Where previous studies have shown the role of incivility in exacerbating stressors and strains among health care workers [57], this study shows how collegial civility and respect can create conditions in which servant leadership can enable well-being to thrive. When there is low experienced WPCC, the influence of servant leadership on follower EWB is constrained. Together these contributions build an argument for the positive role of work on our general well-being. The work context, through leadership behaviors and a civil work climate, influences domain-free EWB illustrating that these functional elements, such as personal growth, environmental mastery, and positive relationships, are particularly pervious to external influences and contextual resources [78,79]. 

Third, where previous studies have linked job performance to psychological well-being as an affective construct [27], this study extends this to psychological well-being in terms of full functioning. Mental health care workers who experienced high levels of EWB also reported higher levels of task performance, innovative work behavior, and work engagement. In line with other findings [54], this study suggests a spill-over effect of work-related relational and contextual factors influencing domain-free EWB, which spirals back into positive effects within the work domain, particularly in the form of work engagement and task performance. This provides further evidence for the value of enhancing domain-free EWB within a work context as it not only benefits work-related outcomes, such as performance and work engagement, but it also contributes to the societal benefit by, alongside hedonic well-being, enhancing eudaimonic components of full functioning, psychological well-being across domains [78]. 

Finally, over and above servant leadership and eudaimonic well-being being directly related to the three workplace outcomes, we further showed that servant leadership was indirectly related to the work outcomes of task performance and work engagement, through EWB, when WPCC was high. This finding suggests that a civil work climate is a considerable factor in enhancing the impact of servant leadership, not only on employee well-being but also on workplace outcomes such as performance and work engagement. In light of this, EWB appears to function as a mechanism explaining the link between job resources and goal-oriented, agential workplace outcomes such as task performance and work engagement. In terms of the COR theory these results seem to support the categorization of different contextual resources according to their functions [50]. The macro resource, as WPCC, functioned as a boundary condition and significantly enabled, or restrained, the ability of servant leadership to impact both well-being and work outcomes. This suggests that contextual resources behave uniquely in their role, not only as motivating or impairing resource gain, but as boundary conditions to these processes. This aligns with the proposition by Grant et al. [30] who claim that managing trade-offs between different dimensions of well-being and the achievement of organizational outcomes may require managerial practices which add value to long-term, broader well-being, and good functioning.

### 4.1. Limitations and Suggestions for Future Studies

A number of limitations in this study need to be considered. First, data were obtained using self-report measures, which carries the risk of common method variance [80]. However, the use of confirmatory factor analysis and the fact that our results aligned consistently with the relevant theory suggest that this impact was limited. Although EWB is difficult to measure other than through self-report, future studies could consider using multiple sources or time-lags. Second, as this study used a cross-sectional design we cannot conclusively claim the effect of a resource gain spiral between servant leadership, EWB, and positive work outcomes, nor regarding the empowerment of this gain by a civil work climate. Although a meta-analysis by Nielsen et al. [81] suggested that the relationship between resources and well-being was not significantly different between cross-sectional and longitudinal studies, it is suggested that a more ambitious longitudinal design would enhance our understanding of whether this dynamic effect holds true over time. 

Finally, the effect sizes of the relationships in this study are rather small. We suspect this could partially be explained by the fact that the scale of psychological well-being [9] is a domain-free measure of EWB and as such perhaps does not fully capture well-being elements particular to work. Future studies could consider the use of measures designed to elicit EWB in a work context [82,83] or by using separate measures for components such as meaningful work [84]. Recognizing that both hedonic well-being and EWB contribute to the flourishing and performance of employees, future studies could consider exploring the dynamic relationship [11] between hedonic well-being and EWB, as stated, and work-related outcomes. Additionally, contextual variables such as challenging working conditions, which have been shown to elicit work engagement [58], or the outcome of quality and safety of care in a health care setting [1], could add value in understanding under which conditions and to what effect EWB is relevant in a work context. It is not only leadership or organizational factors that shape well-being, individual behaviors can also be taken into consideration. Future studies could consider the influence of EWB through its separate dimensions, capturing specific behaviors of individual employees, such as job crafting, which has been linked to increased engagement and decreased burnout [85].

### 4.2. Practical Implications

With the emphasis on servant leaders, in a civil work context, this study offers a concrete avenue and contextual condition under which organizations can influence and shape the EWB of their employees. If aspiring to the mutual gain of both organizational outcomes and employee well-being, it is recommended to select and train leaders in servant leadership, with a particular focus on empowering others, helping subordinates use skills to succeed, ethical behavior, and the value of relationships [72]. Second, both leadership and HR policies and practices can encourage a civil work environment which actively curbs rude, angry behavior and promotes respectful treatment among co-workers. This study gives practical insight into some of the ways in which a psychosocial safety climate could be enacted within an organization [86]. First, by leaders translating these policies and practices, designed to protect workers, into servant leader behaviors such as modeling appropriate behavior, affirming and encouraging others or being open and accountable to others [55]. Second, engaging in dialogues to create good relational habits in the organization, including a focus on integrity and ethics, are important avenues towards creating conditions of workplace civility in which EWB will thrive. Particularly within health care organizations, where ethical behaviors and adherence to codes of conduct are essential to providing professional services, it is likely that this enhances a safe, healthy work environment advantageous to the patient, as well as employee both within and outside of the work domain. Since we know that servant leaders are especially able to model and stimulate a safe working culture [23] this affirms the role of servant leadership in the future of health care [19,20]. Finally, there is increasing recognition of the spill-over between work and private domains, and it seems that encouraging the development of EWB through engaging in meaningful hobbies or learning new skills, even outside of the work domain, would spiral into work-related benefits. 

## 5. Conclusions

This study emphasizes the value of placing EWB centrally within the organizational context. Many organizations focus on measuring employee engagement or short-term happiness in the form of job satisfaction, but neglect to determine avenues to well-being offered by eudaimonic elements such as positive relationships, meaningful work, and the use of strengths. This study provides insights into the contextual and workplace factors, in the form of servant leadership and workplace civility, which actively shape the good functioning of employees, not only within their work context, but in psychological good functioning across individual, organizational, and societal domains.

## Figures and Tables

**Table 1:** Means, standard deviations, and correlations for all study variables.

	Variables	M	SD	1	2	3	4	5	6	7	8	9	10
1	Gender	1.72	0.45	-									
2	Age	46.8	11.9	−0.21 **	-								
3	Education	5.70	1.12	−0.01	−0.29 **	-							
4	Work hours/week	30.4	7.65	−0.37 **	−0.07	0.26 **	-						
5	Tenure	16.1	12.1	−0.06	0.63 **	−0.35 **	−0.19 **	-					
6	Servant leadership	2.92	0.81	−0.11 *	−0.13 *	0.01	0.1	−0.12 *	-				
7	Workplace civility climate	4.28	0.80	−0.03	0.08	0.01	−0.07	0.04	0.24 **	-			
8	Eudaimonic well-being	4.11	0.44	0.04	0.08	0.05	0.02	0.06	0.12 *	0.04	-		
9	Task performance	3.53	0.69	−0.07	−0.02	−0.07	−0.10	−0.02	0.22 **	0.11	0.39 **	-	
10	Innovative work behavior	2.84	0.68	−0.18 **	−0.15 **	0.03	0.27 **	−0.15 **	0.31 **	−0.05	0.14 *	0.15 **	-
11	Work engagement	3.67	0.73	0.03	–0.04	0.01	0.06	−0.02	0.45 **	0.19 **	0.39 **	0.40 **	0.26 **

*Note.* * *p* < 0.05 level ** *p* < 0.001 (two-tailed).

**Table 2:** Moderation of WPCC on the relationship between servant leadership and EWB.

Variables	Step 1				Step 2			
	*B*	*SE (B)*	*t*	*p*	*B*	*SE (B)*	*t*	*p*
Servant leadership (SL)	0.07	0.03	2.17	0.03	0.06	0.03	1.99	0.05
WPCC	0.02	0.03	0.69	0.49	0.06	0.03	1.64	0.10
								
SL × WPCC					0.14	0.04	3.84	<0.001
*R* ^2^	0.02				0.06			
*F*	2.37 ^†^				6.57 ***			
*ΔR* ^2^					0.05 ***			
*ΔF*					14.76 ***			

*Note:* WPCC: Workplace civility climate; EWB: Eudaimonic well-being; SL: Servant leadership. *** *p* < 0.001; ^†^ *p* < 0.10 (two-tailed).

**Table 3:** Results of moderated mediation analysis: The relationship between servant leadership and work outcomes as mediated by EWB and moderated by WPCC.

Predictor	Outcome							
	EWB		Task Performance		Innovative Work Behavior		Work Engagement	
	B	*p*	B	*p*	B	*p*	B	*p*
Servant leadership (SL)	0.06	0.05	0.14	<0.01	0.23	<0.01	0.35	<0.01
WPCC	0.06	0.10	0.10	0.04	−0.13	<0.01	0.07	0.16
Interaction SL × WPCC	0.14	<0.01	0.15	<0.01	−0.13	0.02	−0.03	0.60
EWB			0.53	<0.01	0.23	<0.01	0.57	<0.01
	R² = 0.06		R² = 0.21		R² = 0.21		R² = 0.32	
	F = 6.57		F = 20.14		F = 10.15		F = 36.72	
	*p* < 0.01		*p* < 0.01		*p* < 0.01		*p* < 0.01	

*Note:* SL: Servant leadership; WPCC: Workplace civility climate; EWB: Eudaimonic well-being. For the outcome variable Innovative work behavior we controlled for age (B = −0.01, *p* = 0.13); gender (B = −0.12, *p* = 0.03; tenure (B = −0.00, *p* = 0.03); work hours (B = 0.02, *p* <0.01).
